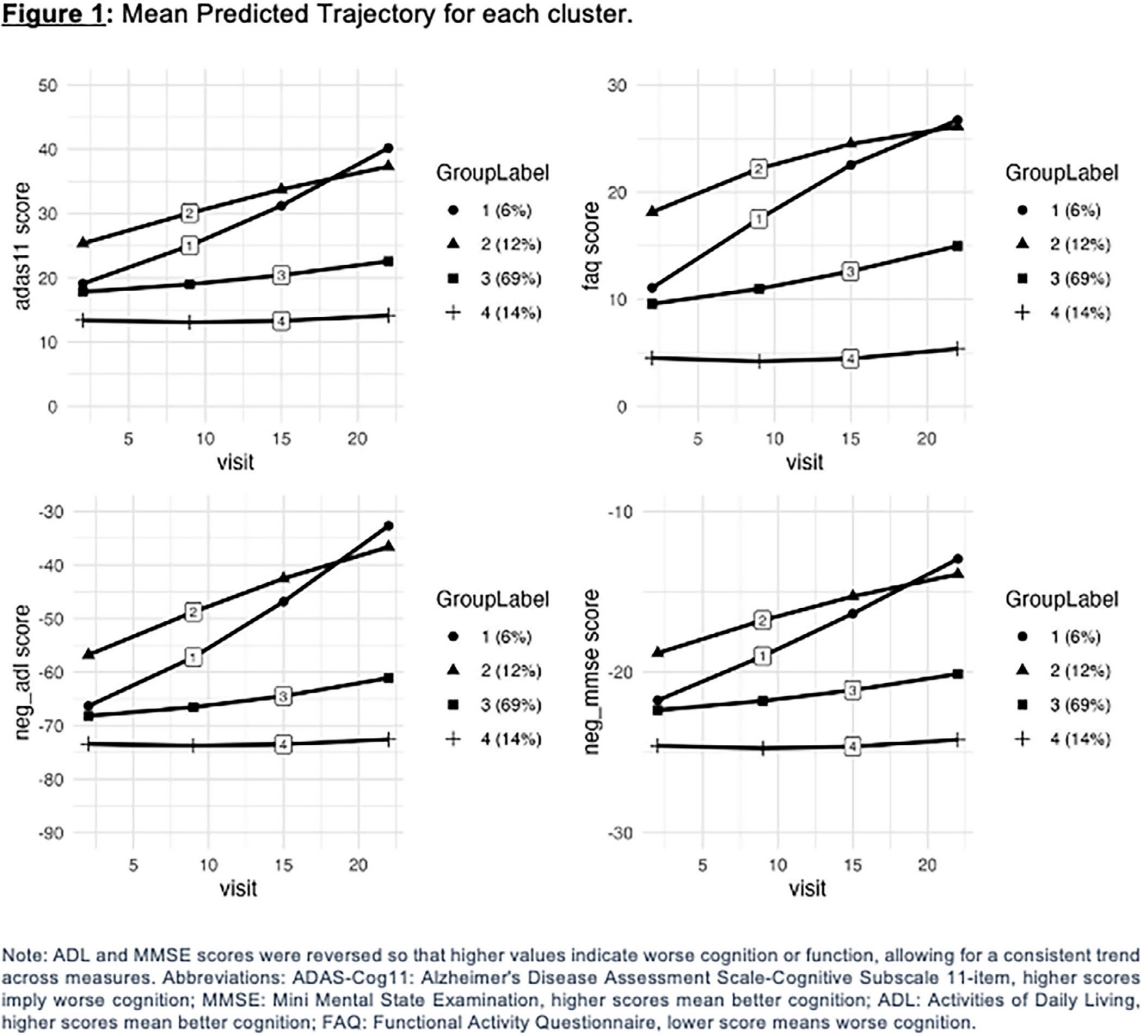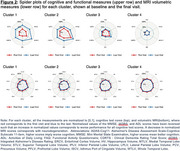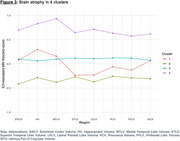# Clustering Cognitive Decline Trajectories in Mild Alzheimer’s Dementia: Insights from the EXPEDITION3 Trial

**DOI:** 10.1002/alz70859_103336

**Published:** 2025-12-25

**Authors:** Jeremy J Lin, Tianchen Qian, Bhargav Teja Nallapu, Richard B. Lipton, Ali Ezzati

**Affiliations:** ^1^ University of California, Irvine, Irvine, CA USA; ^2^ Technical University of Delft, Delft, Zuid‐Holland Netherlands; ^3^ Albert Einstein College of Medicine, Bronx, NY USA

## Abstract

**Background:**

Alzheimer's Disease (AD) progression shows significant heterogeneity, with individuals experiencing varying trajectories of cognitive decline and pathologic changes. Understanding these patterns is critical for tailoring therapeutic strategies and optimizing clinical trial designs. Using a data‐driven approach, we aimed to identify distinct groups with mild dementia based on cognitive and functional trajectories.

**Method:**

We used a latent process mixed model (LPMM) to cluster 924 participants with mild dementia from the placebo arm of the EXPEDITION3 trial. The model incorporated longitudinal cognitive and functional markers (ADAS‐cog11, FAQ, ADL, and MMSE) to identify progression patterns. Identified clusters were further evaluated using demographics and MRI measures from nine brain regions at baseline and at the end of trial.

**Result:**

Four distinct clusters were identified using the LPMM based on Bayesian Information Criterion scores (Figure 1). Cluster 1 (N=47) showed the largest cognitive decline across all measures despite having baseline cognitive scores similar to Cluster 3. Cluster 2 (N=99) had the worst baseline cognitive scores but exhibited a smaller decline compared to Cluster 1. Cluster 3 (N=683), the largest cluster, showed minimal cognitive decline with baseline scores similar to Cluster 1. Cluster 4 (N=95) had the best baseline cognitive scores with minimal cognitive decline. Cluster characteristics based on MRI measures revealed that Cluster 4 had the highest brain volumes across all regions. Cluster 2 exhibited consistently lower volumes across all regions. Clusters 1 and 3 had similar MRI volumes in the entorhinal cortex, hippocampus, and medial temporal lobe, but cluster 1 had reduced volumes in the superior temporal lobe, lateral parietal lobe, precuneus, and prefrontal lobe compared to cluster 3.

**Conclusion:**

Longitudinal cognitive and functional measurements can effectively identify more homogeneous groups among individuals with mild dementia due to AD. These identified groups differ in both baseline and longitudinal progression characteristics, providing insights to the heterogeneity of AD progression. Notably, clusters with similar baseline scores (e.g., Cluster 1 and Cluster 3) displayed markedly different trajectories, highlighting the need to consider longitudinal patterns to inform personalized treatment strategies and to facilitate enrichment designs in future AD clinical trial designs.